# Engaging males to improve nutrition outcomes in young children in Bihar

**DOI:** 10.3389/fnut.2025.1453644

**Published:** 2025-03-13

**Authors:** Putul Thakur, Santosh Akhauri, Narottam Pradhan, Andy Bhanot, Manoj Kumar, Mani Kumar, Neelmani Singh, Sudipta Mondal

**Affiliations:** Project Concern International, Patna, Bihar, India

**Keywords:** male engagement, nutrition, Bihar, minimum dietary diversity, children 6–23 months, human centered design

## Abstract

Poor dietary practices among children aged 6–23 months pose a critical public health challenge, hindering their physical and cognitive development. The National Family Health Survey-5 (NFHS-5) reveals that only 11% of children in this age group consume diets meeting the minimum dietary diversity requirements. To address this, a targeted intervention was designed and implemented to improve dietary diversity in children. This study evaluates the intervention’s impact on enhancing dietary diversity and fostering changes in gender norms, such as increased male participation in nutrition-related decision-making, food procurement, shared childcare responsibilities, and discussions about children’s nutritional needs. This research employed a quasi-experimental design with baseline and endline rounds. The intervention and control blocks were selected from the same district based on matching criteria such as population size, literacy rate, etc. The sample size was determined using a two-sample proportion formula to detect an 9% difference between the intervention and control groups, with a 95% confidence level and 80% statistical power. Household listing identified 1,684 and 1,362 children aged 6–11 months in the intervention and control blocks, respectively. 400 fathers and 400 mothers were randomly sampled from both arms in each survey round. The intervention’s impact was assessed using a difference-in-differences (DID) approach. The results revealed significant improvements in the minimum dietary diversity of children aged 6–23 months (DID coefficient, 21%; *p* < 0.00). At baseline, the intervention and control groups had similar dietary diversity (14% and 13%, respectively), but by endline, the intervention group had significantly improved to 50% compared to 29% in the control group. Knowledge of dietary diversity increased substantially among mothers (DID: 31.3%; *p* < 0.00) and fathers (DID: 15.6%, *p* < 0.00). Collaborative meal planning improved (DID: 9.8%; *p* < 0.00) along with better planning for purchasing vitamin A-rich foods (DID: 28.1%; *p* < 0.00). These findings highlight the effectiveness of engaging men in nutrition programs to support women in child-feeding practices. The intervention improved dietary practices for young children and promoted a gender-inclusive approach. Scaling this program to other regions could enhance child nutrition outcomes and contribute to better child health and development.

## Introduction

Undernutrition among children under 5 years of age is still a public health concern, particularly among 6-23-month-old children in most developing countries. Malnutrition is related to deficiency, excess, or imbalance of energy and other macro-and micronutrients. It comprises varying degrees of under- or overnutrition, leading to changes in body composition, function, and clinical outcomes. Malnutrition is an all-inclusive term that represents all manifestations of poor nutrition, ranging from extreme hunger and undernutrition to obesity (1). Stunting and wasting in children are growth outcomes that result from inadequate dietary intake. Stunting places children at a higher risk of illness and mortality while also leading to delayed cognitive development and diminished intellectual capacity (2). In contrast, wasting impairs immune function, heightens susceptibility to infectious diseases, prolongs the duration and severity of illness, and increases the risk of mortality (3, 4). The India State-Level Disease Burden Initiative reported that malnutrition is the leading cause of death in children under 5 years of age, accounting for 68.2% of all under-five deaths (5).

Bihar is among the Indian states with the highest prevalence of malnourished children. As the third most populous state, Bihar has an estimated population of 100 million, including 12.7 million children under the age of 5 years (6), representing 11% of India’s under-five population. According to the fifth round of the National Family Health Survey (NFHS-5), 43% of under-five children are stunted, and 23% suffer from child wasting in Bihar. Poor dietary practices are a key cause of faltering growth in young children (7–9). Only 11% of the children aged 6–23 months receive an adequate diet (10). Evidence reveals that low dietary diversity significantly interplays with children’s growth failure (11, 12). The World Health Organization (13) defines minimum dietary diversity (MDD) as the consumption of four or more food groups for higher dietary quality and to meet daily energy and nutrient requirements from the seven recommended food groups for children aged 6–23 months. These seven food groups are grains, roots, and tubers; legumes and nuts; dairy products (milk, yogurt, cheese); flesh foods (meat, fish, poultry, and liver/organ meats), eggs, vitamin-A-rich fruits and vegetables, and other fruits and vegetables (WHO and UNICEF).

To address the nutritional challenge, programmatic interventions in these domains have historically focused on women, both as end-users and providers, thus alienating men from the “female domain” of nutrition and reinforcing imbalanced gender roles in this sector (14). However, child nutrition interventions and research can be strengthened by engaging family members, such as fathers, grandmothers, siblings, and extended family members, and by using a family systems framework that explores and reflects family members’ roles, authority, relationships, and communication (15). Evidence demonstrates that families that involve fathers and other key family members, such as mothers-in-law, in nutrition-related activities exhibit greater knowledge and improved practices concerning maternal nutrition and complementary feeding compared to families where nutrition interventions are solely directed toward mothers (16). Moreover, interventions that involve men as agents of positive change are relatively few in number, although research has indicated that men themselves as well as their partners prefer to play a more active role, although societal norms often do not support this (17). There has been recognition that, to improve maternal, infant, and young child nutrition, health structures need to attend to and support fathers, because they play a critical role in providing instrumental and emotional support to mothers and children (18). Engaging men in nutritional interventions often encounter significant barriers. For instance, a study conducted in Rwanda found that most men primarily viewed their roles as providers of financial and material support. However, a smaller subset exhibited more progressive attitudes, expressing a willingness to take on caregiving responsibilities including childcare, meal preparation, and feeding. These findings indicate a shift in traditional gender roles within households (19). Male involvement in infant and young child feeding has been scarcely studied in India (20); however, evidence shows that men are key influencers of infant and young child feeding practices in most rural households (21). To address the existing gaps and challenges in child nutrition, a targeted program was designed using a human-centered design approach and behavioral science principles and implemented in a selected district of Bihar, focusing on improving the dietary diversity of children aged 6–23 months. Thus, the overall aim of the study is to assess the effectiveness of the intervention in improving dietary diversity of young children aged 6–23 months. The primary objective of this study is to evaluate the impact of the program on improving the minimum dietary diversity among children aged 6–23 months in Bihar. Additionally, the secondary objectives are to assess changes in men’s and women’s knowledge of dietary diversity and to explore the extent of male involvement in nutrition-related decision-making, meal planning, food procurement, and shared caregiving responsibilities. These insights aim to guide the design of effective nutrition programs and interventions to address the complex dynamics influencing child nutrition.

## Materials and methods

### Male engagement intervention

The Male Engagement Project was implemented in a block of Samastipur district, Bihar, which was purposefully selected due to Project Concern International’s (PCI) established presence and expertise in the region. The intervention primarily aimed to improve dietary diversity among children aged 6–23 months. Secondary objectives included enhancing parental knowledge about various food groups, fostering male involvement in meal planning and food procurement, promoting shared responsibilities in childcare and feeding practices, and creating an enabling environment by engaging elders and key community influencers to sustain behavior change. The program was developed in six phases, incorporating principles of human-centered design and behavioral science (22). During the initial phase, an extensive review of existing literature was conducted to identify key barriers to dietary diversity among young children. This exercise highlighted limited parental awareness, particularly among fathers, about the importance of dietary diversity and child feeding practices during early childhood. It also revealed the issue of time poverty among mothers, who often struggled to allocate sufficient time to feed their children due to the burden of household chores. Furthermore, while men were typically responsible for food procurement, there was minimal discussion or planning at the household level regarding children’s nutritional needs.

Based on these identified barriers, the program assumed that enhancing parental knowledge and increasing fathers’ engagement in child feeding practices would improve dietary habits for children under 2 years of age. The intervention strategy was guided by the Fogg Behavior Model, which posits that behavior change occurs when three critical elements—motivation, ability, and triggers—align. Motivation refers to the internal drive that influences action, shaped by personal goals or external rewards. Ability encompasses the skills and resources necessary to perform a behavior, while triggers are cues that prompt the desired behavior, provided they are timely and relevant. Evidence suggests that even unmotivated individuals can adopt desired behaviors when provided with effective triggers and that increasing ability can facilitate behavior change, particularly when combined with motivational triggers (23).

Guided by these principles, the intervention employed a range of strategies to achieve its objectives. One key approach involved home visits conducted by Community Resource Person (CRP) couples, who educated parents (both mothers and fathers) on critical topics such as complementary feeding, the importance of diverse food groups, appropriate meal frequency, and meal planning for children. During these visits, the CRP couples utilized structured modules to deliver sessions on dietary diversity, ensuring consistent and comprehensive messaging. These CRPs were married couples from the same community, carefully selected to ensure cultural appropriateness and facilitate open discussions about child feeding practices. Their dual-gender approach enabled more effective engagement with both mothers and fathers. Each CRP couple was assigned a specific catchment area corresponding to a panchayat and was incentivized by the project to sustain their participation and motivation. Further, to enhance the motivation and ability of both mothers and fathers, the intervention utilized videos as educational tools and reinforced key messages through repeated home visits.

As part of the intervention, behavioral triggers such as nutrition calendars and shopping bags featuring images of various food groups were introduced. The calendars were provided to both mothers and fathers of children and placed in their homes. Community Resource Persons (CRPs) trained parents to use stickers on the calendars to track the food groups their children consumed in the past 24 hours. This daily activity helped parents identify gaps in their children’s dietary intake. The shopping bags, featuring images of essential food items, served as reminders for fathers to purchase the necessary items they might otherwise forget while shopping. Additionally, posters with food groups were placed at grocery and cigarette shops, vegetable shops, tea stalls and other locations frequently visited by men. These triggers served as visual reminders or prompts to encourage the purchase of essential food items such as vitamin A-rich fruits, dairy products, eggs, pulses, and vegetables for their children.

**Figure 1:**
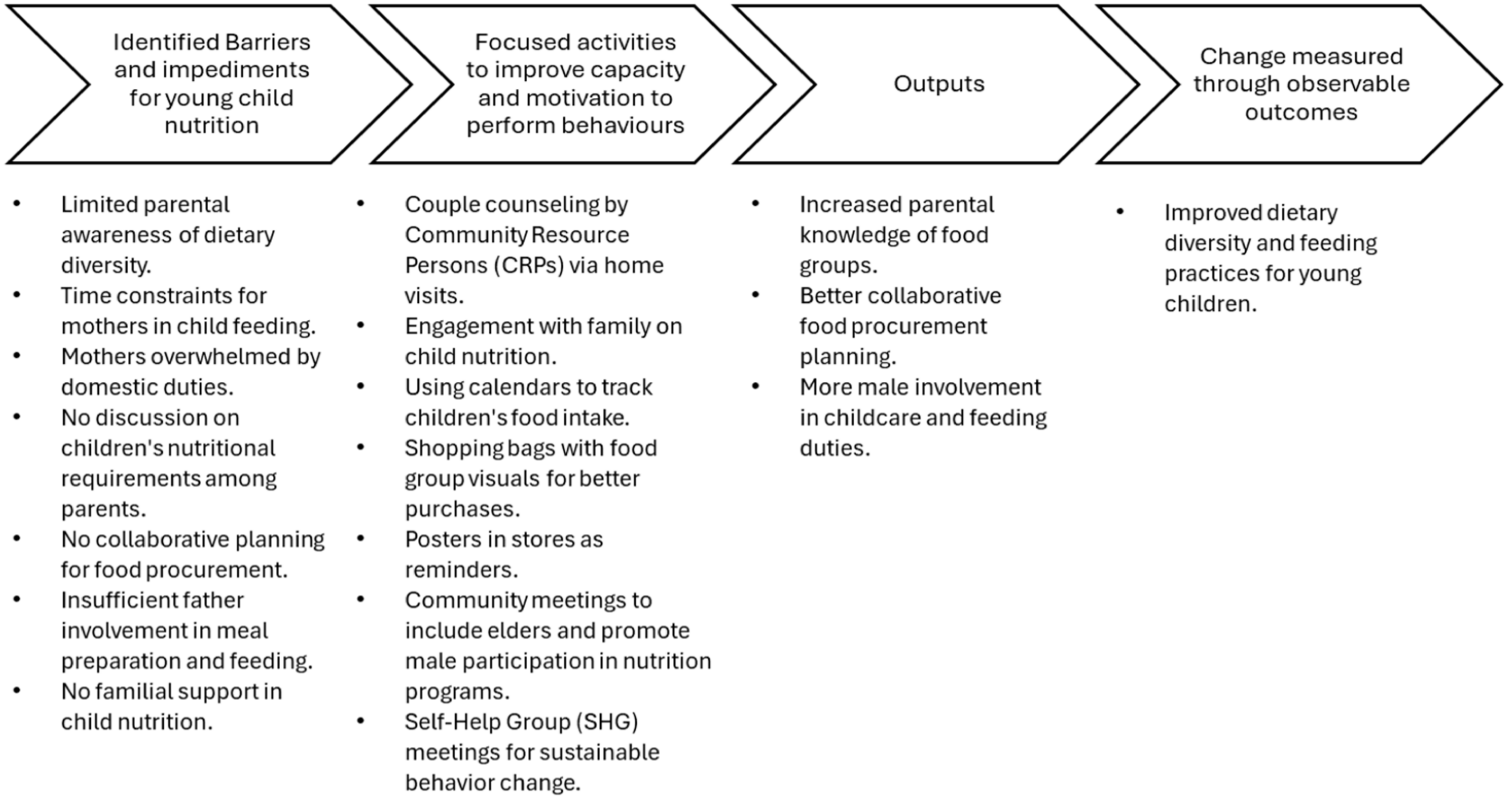
Pathways to change in child feeding practices.

Male groups were established to facilitate discussions on dietary practices, enhance men’s capacity to support improved child feeding and create a sustainable environment for gender-related social norms. Additionally, discussions held during Self-Help Group (SHG) meetings engaged elder women in fostering a supportive environment for promoting and sustaining behavior change. To reinforce sustained adoption of desired behaviors, rewards and recognition were provided to parents who successfully demonstrated these practices. These multifaceted strategies were designed to align motivation, ability, and triggers, as outlined in the Fogg Behavior Model while fostering a supportive community environment for lasting change. The pathways to behavior change adopted in the program are depicted in Figure 1.

### Program evaluation design

The impact evaluation used baseline and endline survey data collected by the Project Concern International (PCI) team. A quasi-experimental design was adopted, with an intervention arm and a control arm. Quasi-experimental design was deemed appropriate for this program evaluation. They are commonly used to assess the effectiveness of interventions, such as nutrition programs, in field settings where random assignment is not feasible (24, 25). The intervention arm participated in the male engagement program. Baseline data were collected in July 2021, and the endline survey was conducted 8 months later, in February 2022.

### Sample size determination and sampling technique

The sample size for this evaluation was calculated using the two-sample proportion test (26). The calculation assumed a 9-percentage point change in the minimum dietary diversity of children aged 6–23 months between the intervention and control arms, with a baseline prevalence of 11%, as reported in NFHS-5 for children in Bihar. The sample size was calculated to achieve 80% statistical power and a 95% confidence level. To account for clustering effects and potential non-response (~10%), the sample size was adjusted to 400 respondents per category per arm. For each survey round, data was collected from 400 mothers and 400 fathers from separate households. This resulted in 800 participants per arm and a total of 1,600 interviews across both rounds (baseline and endline). However, the primary analysis in this paper focuses on data collected from mothers.

A two-stage sampling strategy was adopted to select respondents. The intervention block was purposively selected due to the presence of PCI activities. A control block with a similar socio-demographic profile was identified based on matching criteria, including population size, literacy rate, road connectivity, access to education, and primary occupation, with a ± 10% variation allowed. Subsequently, 15 panchayats were randomly selected within each block. A household listing exercise was then conducted to create a sampling frame of children aged 6–11 months, identifying 1,684 eligible children in the intervention block and 1,362 in the control block. From these frames, 800 households (400 women and 400 men) per arm were randomly selected for baseline interviews. The same list was used for the endline to ensure consistency and comparability, allowing for some overlap between rounds.

### Structured questionnaire and measurement

A structured questionnaire was developed, encompassing five domains: the demographic profile, fathers’ and mothers’ knowledge of minimum dietary diversity, dietary diversity practices for children aged 6–23 months, collaborative planning for food procurement, and male involvement in nutrition-related decision, food purchases, food preparation and sharing responsibilities for child feeding. The questionnaire was designed to align with the study objectives and was thoroughly reviewed by all authors to ensure content validity and relevance. Several domains of the questionnaire were adapted from established tools and guidelines, while certain questions were developed based on evidence from relevant published studies (27–37). Each question was carefully discussed and revised to ensure clarity and conciseness. The tool was double-translated to ensure linguistic and contextual accuracy: first from English to Hindi, followed by back-translation from Hindi to English by an independent translator.

Further, the section on knowledge about different food groups was adopted from previous studies, as it influences dietary practices (34, 35). The knowledge question was designed as a close-ended item with three categorical response options: less than four food groups, four or more food groups, and “do not know.” The question was asked to both mothers and fathers to assess their knowledge of minimum dietary adequacy for their children. Parents who responded with four or more food groups were classified as having knowledge of Minimum Dietary Diversity (MDD) for their children.

Data on children’s dietary intake over the previous 24-hours period were obtained from mothers. The questionnaire was adapted based on the FANTA guidelines for calculating minimum dietary diversity scores, incorporating insights from previous studies by various researchers (30, 32). Information on 16 food items, that were consumed by the child a day before, was categorized into 7 groups as follows: (a) grains, roots, tubers and plantains; (b) pulses (beans, peas, lentils), nuts and seeds; (c) dairy products (milk, infant formula, yogurt, cheese); (d) flesh foods (meat, fish, poultry, organ meats); (e) eggs; (f) vitamin-A rich fruits and vegetables; and (g) other fruits and vegetables (37). Further, the dietary diversity score (DDS) was calculated using seven food groups, as specified by the FANTA guidelines (38). Children consuming food from at least four food groups out of seven food groups were considered to meet the Minimum Dietary Diversity (MDD) standard (WHO and UNICEF).

Further, questions on collaborative meal planning and procurement were adapted from studies conducted on adult populations (28, 36). Meal planning and procurement are potential strategies to alleviate time poverty and improve dietary diversity and diet quality. However, there is a paucity of research focusing on these factors in relation to young children. Mothers were asked about how often their families visit the market to buy food for their children aged 6–23 months, as well as how they collaborate and discuss purchases with their spouses. Responses to the questions included: always, sometimes, rarely, and never. Participants who responded with “always” or “sometimes” were classified as collaboratively planning, while those who responded “rarely” or “never” were classified as not planning before purchasing food items for children. Subsequently, respondents were asked about the specific food items they plan to purchase. Further, food items were categorized into food groups as recommended in the FANTA guideline (38).

Further, the last domain of the tool included four statements using a 5-point Likert scale to assess women’s perceptions of changes in men’s roles in enhancing dietary diversity for children. These statements evaluated men’s participation in supporting their wives’ nutrition-related decisions, sharing child-feeding responsibilities, purchasing food items, and discussing children’s nutritional needs. Responses were coded as follows: 4 for “strongly agree,” 3 for “agree,” 2 for “disagree,” 1 for “strongly disagree,” and 0 for “neutral.” Women who responded “strongly agree” reported that their husbands actively participated in purchasing food items, supported nutrition-related decisions, engaged in discussions about nutritional requirements, and shared responsibilities for meal preparation and child feeding. The questions were developed based on existing literature on male engagement in child-feeding practices (27, 28, 31, 33).

The study tool was pretested to evaluate its applicability, clarity, relevance, reliability, and cultural appropriateness prior to data collection. Pretesting involved approximately 5% of the sample population in a non-intervention area. Field observations during pretesting were documented, resulting in amendments to the study questionnaire, including improvements in translation and skip logic. Data collection was conducted using hand-held devices. Supervisors and data collectors underwent comprehensive training on data collection procedures to ensure accuracy and consistency. The study was approved by the Ethics Committee of SIGMA Institutional Review Board (New Delhi) with the reference number 10067/IRB/20–21. Verbal consent was taken from the respondents prior to the interview, and the IRB approved taking the participants’ verbal consent during the interviews. All interviews are conducted with adult participants only, and none of the minors are interviewed.

### Outcome measures

The program’s core strategy focuses on improving the dietary practices of children aged 6–23 months by adopting a gender-centric approach that emphasizes engaging men in nutrition-related practices. It promotes collaborative discussions and planning for food procurement, encourages husbands to actively support their wives in meal preparation, and fosters shared responsibilities for cooking and feeding children. The study evaluates its impact through four outcome indicators: (1) Minimum Dietary Diversity (MDD) among children aged 6–23 months, (2) mothers’ and fathers’ knowledge about MDD, (3) collaborative planning for food purchases, and (4) husbands’ participation in nutrition-related decision-making, food procurement, and discussions about children’s nutritional needs. The methodology, including key questions, response categories, and coding, has been detailed in the previous section.

### Statistical analyses

Descriptive analysis was carried out to ascertain the distribution of different parameters in the study population, and all analyses were conducted using Stata 15.0. Quantitative variables were examined for normality. The chi-square test and Fisher’s exact test were used to compare the baseline characteristics of the intervention and control groups, as well as the endline characteristics in both rounds. Difference-in-differences (DID) was applied to assess the impact of program exposure on the consumption of a minimum diversified diet, knowledge of dietary diversity, collaborative planning for food procurement, and male participation in supporting wives with food preparation and feeding practices. The analysis included socio-demographic factors as covariates to account for potential confounders, including age, education level, household size, social group (Scheduled Caste/Scheduled Tribe, Other Backward Caste, General), and child age. Moreover, the baseline scores for each outcome were included as covariates in the DID model to adjust baseline differences between the intervention and control groups. Controlling for these variables in the Difference-in-Differences (DID) regression model allowed for a more precise estimation of the intervention’s impact, isolating its effects from pre-existing differences between groups. The DID method is appropriate for assessing the impact of the program, as it compares the outcome measures of the intervention and control groups across baseline and endline periods (39).

## Results

### Sociodemographic characteristics of study participants

The demographic characteristics of both the intervention and control groups remained consistent across baseline and endline periods, as illustrated in Table 1. The sample predominantly consists of Hindus, comprising 87% (baseline-84.4%, endline-89.2%) in the intervention group and 90% (baseline-88.8%, endline-91.0%) in the control group. Scheduled caste and scheduled tribe (SC/ST) households accounted for 31% (baseline-33.4%, endline-29.0%) in the intervention arm and 35% (baseline-34.2%, endline-35.0%) in the control arm. The majority of households reported a nuclear family structure, with 39% (baseline-36.0%, endline-41.6%) in the intervention group and 40% (baseline-39.0%, endline-41.0%) in the control group. Additionally, most households had more than 5 members in both groups. The median age of female respondents ranged from 24 to 25 years in both groups, indicating no significant variation within the sample. A substantial proportion of female respondents had completed schooling up to the 9th standard across periods in both groups, comprising 71% (baseline-73.9%, endline-55.1%) in the intervention group and 70% (baseline-72.0%, endline-69.9%) in the control group. High rates of female unemployment persisted throughout both periods and in both groups, reflecting entrenched gender norms assigning women primary responsibility for childcare and household duties. Approximately 50% of women in the sample had three or more children, indicating a high total fertility rate. No disparities were observed in the gender or age distribution of children across periods and groups.

**Table 1:** Socio-demographic characteristics of women participants in baseline and endline.

	Intervention			Control		
Characteristics	Baseline (*N* = 398)	Endline (*N* = 341)	*p*-value	Baseline (*N* = 418)	Endline (*N* = 354)	*p*-value
Religion	%	%		%	%	
Hindu	84.4	89.1	0.060	88.8	91.0	0.314
Non-Hindu	15.6	10.9		11.2	9.1	
Caste						
SC/ST*	33.4	29.0	0.144	34.2	35.0	0.964
OBC**	59.1	65.7		60.3	59.3	
General	7.5	5.3		5.5	5.7	
Type of family						
Nuclear	35.9	41.6	0.112	39.0	41.0	0.578
Joint	64.1	58.4		61.0	59.0	
No. of household members						
Upto 4 members	18.1	19.4	0.306	13.4	20.3	0.034
5–6 members	37.9	42.2		40.0	35.9	
more than 6 members	44.0	38.4		46.6	43.8	
Age of respondent						
Median age of women	24 Years	25 Years		24 Years	24 Years	
18–22 Years	36.7	34.3	0.053	37.8	30.5	0.1
23–25 Years	34.2	28.5		33.3	38.1	
Above 25 Years	29.2	37.2		29.0	31.4	
Education attainment						
No schooling	38.2	27.9	0.009	31.1	30.8	0.824
Up to 9th	35.7	44.3		40.9	39.3	
Above 9th	26.1	27.9		28.0	29.9	
Occupation of mother						
Agri-based	4.3	10.9	0.001	1.7	7.1	0.000
Non-Agri-based	3.0	5.9		5.0	6.5	
Salaried	1.5	2.6		0.2	0.9	
Unpaid family business	3.0	3.5		1.0	3.1	
Unemployed	88.2	77.1		92.1	82.5	
Parity						
1–2 Children	53.5	50.4	0.404	50.0	50.9	0.814
3 or more Children	46.5	49.6		50.0	49.2	
Sex of the youngest child (6–23 months)						
Boy	55.0	51.0	0.277	51.7	49.4	0.535
Girl	45.0	49.0		48.3	50.6	
Age of the youngest child(6–23 months)						
6–8 months	26.6	0.9	0.000	24.6	0.3	0.000
9–11 months	25.9	2.9		29.7	0.6	
12–23 months	47.5	96.2		45.7	99.2	

*SC/ST, Scheduled Caste/ Scheduled Tribe, **OBC, Other Backward caste.

### Dietary diversity among children

The 24-hours recall of dietary diversity score findings showed a significant improvement after the intervention. Child dietary diversity improvements were significantly higher among respondents in the intervention arm compared to those in the control arm. Dietary diversity among children increased from 13% to 29% in the control arm, while it rose from 14% to 50% in the intervention arm (Table 2). The average treatment effect of the program on dietary diversity among young children was 21% (DID coefficient: 21%; *p*-value <0.001). This indicates that the intervention contributed to improving dietary diversity among children aged 6–23 months.

**Table 2:** Net effect of the program on improving dietary practices.

	Baseline		Endline		St. Err.	*t*-value	DID Coef.	*p*-value
Dietary practices	Intervention	Control	Intervention	Control				
Minimum dietary diversity- Children	14%	13%	51%	29%	0.042	4.84	21%***	0.000
Consumption of grains, white/ pale starch, roots, tubers and plantains	75%	79%	98%	94%	0.035	2.00	7%**	0.046
Consumption of beans, peas, lentils, nuts and seeds	59%	59%	82%	79%	0.047	0.82	4%	0.414
Consumption of dairy Products	43%	46%	66%	54%	0.051	2.89	15%***	0.004
Consumption of flesh foods (meat, fish, poultry, organ meat)	1%	1%	14%	7%	0.023	3.23	7%***	0.001
Consumption of eggs	4%	2%	19%	5%	0.026	4.59	12%***	0.000
Consumption of vitamin-rich fruits and vegetables	14%	6%	24%	12%	0.035	1.04	4%	0.301
Consumption of other fruits and vegetables	17%	20%	57%	36%	0.045	5.49	25%***	0.000

Means and standard errors are estimated by linear regression. **Inference: ***p < 0.01; **p < 0.05; *p < 0.1.

Table 2 summarizes the program’s net effect on the consumption patterns of various food groups among children aged 6–23 months. A high proportion of children consumed grains, roots, tubers, and plantains, with rates increasing from 79% and 75% at baseline to 94% and 98% at endline in the control and intervention groups, respectively. A significant improvement was observed in the consumption of grains and tubers, with a net effect of 7% (*p*-value: 0.046). The consumption of pulses, beans, nuts, and seeds increased from 59% at baseline in both groups to 79% and 82% at endline in the control and intervention groups, respectively, with a net effect of 4%; however, the change was not statistically significant.

Egg consumption remained low, increasing from 2% and 4% at baseline to 5% and 19% at endline in the control and intervention arms, respectively. This change was significant in the intervention arm, with a net effect of 12% (*p*-value <0.001). While the consumption of vitamin A-rich fruits and vegetables improved, the change was not statistically significant between survey rounds. However, the consumption of other fruits and vegetables rose significantly, from 20% to 30% in the control arm and from 17% to 57% in the intervention arm, with a net effect of 25% (*p*-value <0.001). Similarly, the consumption of milk and dairy products increased from 46% to 54% in the control arm and from 43% to 66% in the intervention arm, with a net effect of 15% (p-value <0.001), indicating a positive impact of the program intervention.

### Improvement in fathers’ and mothers’ knowledge of child dietary diversity

The difference-in-differences (DID) analysis confirmed the significant impact of the program on improving knowledge among both men (fathers) and women (mothers) regarding the consumption of at least 4 food groups by young children in a day (Table 3). Knowledge among mothers increased from 6% to 12.7% in the control arm and from 6.5% to 44.6% in the intervention arm. The unadjusted effects are statistically significant for knowledge of the consumption of different food groups in a day (Table 3; DID coefficient- 31.3%; *p*-value<0.00). Knowledge among fathers increased from 4.2% to 7.8% in the control arm and from 3.8% to 23% in the intervention arm. The unadjusted effects are statistically significant for knowledge among men (DID coefficient- 15.6%; *p*-value<0.00).

**Table 3:** Knowledge of minimum dietary diversity among men and women study participants.

	Baseline		Endline		St. Err.	*t*-value	DID Coef.	*p*-value
	Intervention %	Control%	Intervention%	Control%				
Women’s knowledge of four or more food group consumption for children aged 6–23 Months	6.5	6	44.6	12.7	0.035	8.99	31.3***	0.000
Men’s knowledge of four or more food group consumption for children aged 6–23 Months	3.8	4.2	23	7.8	0.030	5.17	15.6***	0.000

Means and standard errors are estimated by linear regression. *Significant difference between baseline and endline (*p* = 0.05). **Significant difference between baseline and endline (*p* = 0.001). ***Significant difference between baseline and endline (*p* < 0.001).

Table 4 presents significant advancements in maternal knowledge regarding the consumption of specific food groups. The Difference-in-Differences (DID) analysis indicates substantial increases in knowledge, with a DID coefficient of 23.6% (*p* < 0.000) for flesh food consumption, 19.0% (*p* < 0.000) for egg consumption, 18.2% (*p* < 0.000) for vitamin A rich foods, and 10.0% (*p* < 0.05) for other fruits and vegetables.

**Table 4:** Mothers’ knowledge of food group consumption for children aged 6–23 months.

	Baseline		Endline		St. Err.	*t*-value	DID Coef.	*p*-value
Knowledge among women about MDD	Intervention %	Control %	Intervention %	Control %				
Grains, white/ pale starch, roots, tubers and plantains	99.7	99.8	98.2	98.9	0.009	0.67	0	0.500
Beans, Peas, Lentils, nuts and seeds	92	94	94.7	92.7	0.026	1.60	0.4	0.110
Dairy Products	80.9	79.9	80.9	78.8	0.041	0.27	0.1	0.785
Flesh Foods (meat, fish, poultry, organ meat)	15.6	19.1	38.4	18.4	0.042	5.61	23.6***	0.000
Eggs	14.8	13.6	35.5	15.3	0.040	4.79	19.0***	0.000
Vitamin A rich fruits and vegetables	17.3	14.1	45.2	23.7	0.043	4.28	18.2***	0.000
Other fruits and vegetables	56.3	49	83.6	66.4	0.048	2.07	10.0**	0.039

Means and standard errors are estimated by linear regression. *Significant difference between baseline and endline (*p* = 0.05). **Significant difference between baseline and endline (*p* = 0.001).

Table 5 highlights the improvement in fathers’ knowledge regarding the daily consumption of different food groups by young children. The findings indicate a significant enhancement in awareness, with knowledge of flesh food and meat consumption increasing by 15.5% (*p* < 0.001), egg consumption by 20.3% (*p* < 0.001), and vitamin A-rich food consumption by 16.6% (*p* < 0.001).

**Table 5:** Fathers’ knowledge of food group consumption for children aged 6–23 months.

	Baseline		Endline		St. Err.	*t*-value	DID Coef.	*p*-value
Knowledge among men about MDD	Intervention%	Control %	Intervention%	Control%				
Grains, white/ pale starch, roots, tubers and plantains	96.7	98.5	97.1	96.8	0.018	1.15	2.1	0.252
Beans, Peas, Lentils, nuts and seeds	85.8	91	86	85.5	0.038	1.51	5.7	0.132
Dairy Products	76.9	73.3	77	77.5	0.048	0.86	−4.1	0.387
Flesh Foods (meat, fish, poultry, organ meat)	9.1	7.5	27.6	10.4	0.036	4.30	15.5***	0.000
Eggs	10.9	11.1	32.5	12.4	0.039	5.15	20.3***	0.000
Vitamin A rich fruits and vegetables	11.2	9.3	33.7	15.3	0.040	4.15	16.6***	0.000
Other fruits and vegetables	45.9	44.4	73.7	63.6	0.054	1.58	8.6	0.114

Means and standard errors are estimated by linear regression. *Significant difference between baseline and endline (*p* = 0.05). **Significant difference between baseline and endline (*p* = 0.001).

### Collaborative meal planning and procurement

Mothers were asked about which household members were primarily responsible for visiting marketplaces to procure food. They were also asked about the frequency of these visits and the extent of collaborative planning with male household members prior to food procurement. The DID analysis confirmed that both fathers and mothers planned food purchases for their children (Table 6). Planning before food procurement increased from 6.5% to 25.4% in the control arm and from 9% to 37.8% in the intervention arm, which resulted in a net effect of 9.8% (*p*-value <0.00). These findings suggested that the program effectively enhanced collaborative planning and discussions among mothers, fathers, and caregivers prior to visiting marketplaces. Additionally, the provision of tote bags for carrying vegetables served as practical reminder to prioritize the purchase of essential food items for children.

**Table 6:** Women’s responses on meal planning and procurement of food items for children aged 6–23 months.

	Baseline		Endline		St. Err.	*t* value	DID Coef.	*p* value
Indicator	Intervention %	Control %	Intervention %	Control %				
Planned food purchases for children aged 6–23 months prior to Market Visits	9.0	6.5	37.8	25.4	0.038	2.58	9.8***	0.010
Planned to purchase Vitamin A rich fruits and vegetables like mango, papaya, pumpkin, green leafy vegetables, spinach, carrot	17.6	15.3	35.7	5.2	0.074	3.77	28.1***	0.000
Planned to purchase pulses and nuts	18.8	26.4	35.2	17.6	0.083	3.02	25.2***	0.003
Planned to purchase Grains, roots and tubers	3.5	25	15.2	15.0	0.067	3.23	21.7***	0.001
Planned to purchase other fruits and vegetables like apple, banana, guava, bottle guard, cauliflower	38.8	50.0	79.0	64.1	0.088	2.97	26.2***	0.003
Planned to purchase Milk and Milk products	24.7	37.5	16.2	11.8	0.074	2.32	17.2**	0.021
Planned to purchase Eggs and Poultry products	2.4	5.6	18.6	7.2	0.058	2.50	14.6**	0.013
Planned to purchase Meat and Flesh food	1.2	0	11.4	0.7	0.041	2.35	9.6**	0.019

Means and standard errors are estimated by linear regression. *Significant difference between baseline and endline (*p* = 0.05). **Significant difference between baseline and endline (*p* = 0.001). ***Significant difference between baseline and endline (*p* < 0.001).

A significant improvement was observed in the purchase of Vitamin A-rich food items, such as mango, papaya, pumpkin, and green leafy vegetables (DID coefficient: 28.1%; *p*-value <0.00); nuts (DID coefficient: 25.2%; *p*-value <0.00); other fruits and vegetables (DID coefficient: 26.2%; *p*-value <0.00); milk and dairy products like cheese (DID coefficient: 17.2%; *p*-value <0.05); eggs (DID coefficient: 14.6%; *p*-value <0.05); and flesh and meat (DID coefficient: 9.6%; *p*-value <0.05; Table 6). Vitamin A-rich vegetables and fruits, milk and milk products, eggs, and flesh foods are vital for the growth and development of young children. Due to their perishable nature, these food items require frequent purchases throughout the week. The findings, as presented in Table 6 reveal that both men and women actively plan to procure these items and incorporate them into their children’s diet.

### Men’s role in supporting their wives to enhance dietary diversity for children aged 6–23 months

The program focused on directly engaging men (fathers) to improve their understanding of food groups and support their wives in child-feeding practices. To assess the program’s impact on men’s behavior, women responded to four statements on a 5-point Likert scale. These statements evaluated women’s perceptions of their husbands’ involvement in nutrition-related decision-making, purchasing food items for children, sharing child-feeding responsibilities, and discussing their child’s nutritional needs. The DID analysis demonstrates an improvement in male engagement among respondents in the intervention group. Women reported that male participation in nutrition-related decisions for their children improved (DID coefficient: 11.1%; *p*-value <0.00). Husbands consistently remembered to include food items for their children when purchasing household groceries (DID coefficient: 7.1%; *p*-value <0.10). Male participation in feeding their children (DID coefficient: 16.5%; *p*-value <0.10) and discussing their children’s food requirements over the past 3 months (DID coefficient: 23.3%; *p-*value <0.10) further supports the evidence of increased male engagement in child-feeding practices within the intervention area (Table 7).

**Table 7:** Women’s perception of men’s participation in child feeding practices.

	Baseline		Endline		St. Err.	*t*-value	*p*-value	DID Coef.
	Intervention %	Control %	Intervention %	Control				
My husband supports me in making nutrition-related decisions or requirements for my child aged 6–23 months.	70.6	80.1	96.5	94.9	0.035	3.13	0.002	11.1***
My husband remembers to buy the food requirements for my child under 2 years every time, he purchases groceries and food items for the household	65.1	69.1	90.6	87	0.042	1.83	0.068	7.7*
My husband regularly shares the responsibility of feeding our child under 2 years	58	66.5	88.6	80.5	0.045	3.71	0.000	16.5***
My husband and I discussed the food requirements of our child under 2 years in the last 3 months	62.1	72.7	90.6	78	0.043	5.37	0.000	23.3***
Separate food preparation for children 6–23 months old	14.8	12	33.1	17.8	0.040	3.15	0.002	12.5***

Means and standard errors are estimated by linear regression. *Significant difference between baseline and endline (*p* = 0.05). **Significant difference between baseline and endline (*p* = 0.001). ***Significant difference between baseline and endline (*p* < 0.001).

Additionally, the findings reveal an increase in the practice of separate cooking for children (DID coefficient: 12.5%; *p*-value <0.00; Table 7).

## Discussion

This study evaluated the effectiveness of the intervention in engaging men to enhance the minimum dietary diversity among children aged 6–23 months. While male involvement in child nutrition has been widely studied in African countries, there is a notable gap in research addressing this topic within the Indian context. Given the cultural and familial dynamics in India, this research provides valuable insights into how encouraging male participation in household nutrition can significantly enhance dietary diversity, which is crucial for the healthy growth and development of young children. Understanding the role of fathers and male caregivers in India could offer new avenues for public health interventions to address child malnutrition. Studies from other parts of the world reveal that Men’s nutrition knowledge is associated with children’s dietary outcomes (40) and men often play a large role in shaping decision-making, food choices and time availability within a household (27). However, men play a limited direct role in childcare and feeding, rather it is seen as a woman’s responsibility (36). Women often experience time poverty due to the burden of household chores, which can lead to poor child-feeding practices (41). Male engagement in childcare responsibility and feeding along with familial support may lead to improved dietary practices (42). There is limited evidence on targeted intervention strategies that effectively engage men in child nutrition programs, as well as on the barriers and facilitators that shape their participation. This study’s male engagement program was developed using a Human-Centered Design Approach to address these gaps. The program was piloted in selected regions of Bihar, demonstrating its efficacy and potential to positively influence men’s involvement in young child feeding practices.

A comparison between the baseline and endline rounds, along with the control groups, unveils five significant findings from the study. Firstly, there was an improvement in the dietary diversity of children aged 6–23 months. A substantial shift occurred in consumption patterns, particularly toward higher intake of pulses, fruits, vegetables, and dairy products. Supporting this finding, a similar study from Tanzania demonstrated that male-headed households exhibit higher dietary diversity among children, women, and the household overall compared to female-headed households (34, 43). Additionally, these findings highlight the critical role of males in believing that men are primarily responsible for decisions regarding food purchases, such as where to buy, pricing considerations, and meal selections, while their involvement in child-feeding practices is minimal (31). Previous programs have predominantly targeted women, especially mothers, neglecting men (32, 44). Despite women’s active participation and awareness of young children’s dietary practices, substantial change has yet to be realized. In the current program, both men and women were jointly addressed during home visits. This fostered collective discussions on the importance of dietary diversity, feeding frequency, and the financial consequences of recurrent illnesses due to malnutrition among young children, encouraging couples to engage in conversations about diversified diets. To monitor these practices, calendars were distributed, and follow-up visits were conducted by the Community Resource Person (CRP) couples. This approach enabled mothers and fathers to provide mutual support in feeding their young children.

Secondly, there was an improvement in knowledge regarding different food groups and the importance of their consumption among both fathers and mothers. This enhancement can be attributed to counseling provided by CRP (Community Resource Person) couples during household visits. These visits were strategically planned with prior appointments to ensure the presence of both parents, facilitating shared learning. CRP couples utilized modules and audio-visual tools to strengthen the impact of their counseling and influence parental behavior, with the videos made available for later review as needed. Interestingly, the improvement in knowledge was more significant among women compared to men. The reasons for this difference suggest further investigation; however, it is likely that men are newer to participating in food-related discussions, whereas women have traditionally been more involved. Sustained engagement with fathers is likely to deepen their knowledge of food and dietary diversity, ultimately leading to better nutritional outcomes for young children. Evidence suggests that men are generally less interested in nutrition-related knowledge compared to women (45); however, their involvement leads to improved nutritional outcomes among children and women (40). Exposure to the program notably improved knowledge among both men and women regarding the consumption of flesh foods, eggs, and vitamin A-rich fruits and vegetables.

Thirdly, the project enhanced meal planning practices prior to food procurement, specifically addressing the nutritional needs of young children. Traditionally, male members of the household are tasked with purchasing food, often without engaging in prior discussions or planning (43). However, this project improved men’s knowledge of various food groups and fostered collaborative discussions between men and women before visiting markets. As a result, there was a notable increase in the procurement of essential food items, including vitamin A-rich fruits and vegetables, pulses, nuts, eggs, and flesh foods. To support the practical application of this knowledge, grocery shops and marketplaces in nearby villages were selected, and signboards displaying messages about different food groups were installed. These visual reminders encouraged men (fathers) to make informed purchasing decisions. The findings align with previous research indicating that when men engage in food procurement, provide financial support, and discuss food needs with their wives, it positively influences food selection and overall household nutrition. Furthermore, the project observed an increase in the consumption of dairy products, highlighting the beneficial effects of enhanced male participation in nutrition planning (43).

Finally, the project successfully promoted greater male involvement in supporting their wives with nutrition-related decision-making, food purchasing, shared responsibilities in child feeding, and discussions about their children’s nutritional needs. A similar qualitative study from Rwanda highlighted that while women are typically responsible for purchasing and preparing food for the family, men retain control over financial resources and decision-making. This dynamic often leaves women feeling disempowered in the face of male-dominated decision-making processes (46).

By fostering collaborative planning and shared responsibilities within households, the program not only increased male support but also empowered women to play a more active role in ensuring optimal child nutrition. This partnership-based approach reinforced women’s decision-making capacity and strengthened their ability to address their children’s nutritional needs effectively.

Given the scarcity of literature and evidence on men’s involvement in child nutrition, especially in India, the Male Engagement Program offers a valuable opportunity. This initiative will aid researchers, policymakers, and program implementers in transforming the nutritional landscape to improve the growth and well-being of young children.

## Conclusion

This study underscores the significant role of male engagement in improving dietary diversity among children aged 6–23 months, a critical public health priority. The intervention demonstrated that enhancing fathers’ and mothers’ nutritional knowledge, encouraging their involvement in meal planning, purchasing, and preparation, and fostering discussions around child nutrition led to better child-feeding practices. The adoption of the Human-Centered Design (HCD) process, grounded in behavioral science principles, was particularly effective in designing the program by identifying and addressing barriers to male engagement in child-feeding practices. Effective strategies included joint discussions among fathers and mothers before food purchases and utilizing feeding calendars. The involvement of Community Resource Person (CRP) couples from within the community was pivotal in educating both mothers and fathers and fostering shared responsibilities for child nutrition. Additionally, tools like shopping bags and display posters showcasing food group images, strategically placed in prominent community locations, encouraged fathers to make diverse food choices for their children. Discussion groups for men, combined with the engagement of female SHGs, played a crucial role in fostering an environment conducive to positive change. These findings offer valuable insights for policymakers and program implementers aiming to enhance child nutrition through inclusive family-centered interventions, contributing to the under-researched area of male involvement in child nutrition in India.

This study has limitations that warrant cautious interpretation of the results. Firstly, this study employed a quasi-experimental design, which, while useful in real-world settings, presents certain limitations. First, the absence of randomization may introduce selection bias, as participants in the intervention and control groups may differ in unobserved ways that affect outcomes. While we used Difference-in-Differences (DID) analysis to adjust for baseline differences, residual confounding may still exist, potentially affecting the interpretation of the intervention’s impact. Secondly, the study assesses male involvement in child feeding and caregiving practices. The reliance on self-reported data from male respondents introduces the possibility of social desirability bias, as men may overstate their participation in these activities due to the perceived positive role they are expected to play in household nutrition. Additionally, cultural norms and gender expectations may shape both reported and actual behaviors, which could limit the generalizability of the findings across diverse contexts. To mitigate this bias to some extent, corresponding data from interviews with women have also been included to provide a more balanced perspective.

Future research should explore the long-term sustainability of male engagement in child nutrition and assess the scalability of this approach in diverse contexts. Overall, this study highlights the importance of inclusive, family-centered nutrition strategies and offers a replicable model for achieving sustainable improvements in child dietary diversity with the potential for broader scaling.

## Data Availability

The datasets generated for this study and used for analysis shall be available on request to the corresponding author.
